# Intratumoral immunosuppression profiles in 11q‐deleted neuroblastomas provide new potential therapeutic targets

**DOI:** 10.1002/1878-0261.12868

**Published:** 2021-01-19

**Authors:** Esther Coronado, Yania Yañez, Enrique Vidal, Luis Rubio, Francisco Vera‐Sempere, Antonio José Cañada‐Martínez, Joaquín Panadero, Adela Cañete, Ruth Ladenstein, Victoria Castel, Jaime Font de Mora

**Affiliations:** ^1^ Laboratory of Cellular and Molecular Biology Health Research Institute Hospital La Fe Valencia Spain; ^2^ Clinical and Translational Research in Cancer Health Research Institute Hospital La Fe Valencia Spain; ^3^ Roche Diagnostics Information Solutions Basel Switzerland; ^4^ Department of Pathology La Fe University Hospital Valencia Spain; ^5^ School of Medicine University of Valencia Spain; ^6^ Data Science Unit Health Research Institute Hospital La Fe Valencia Spain; ^7^ Genomics Unit Health Research Institute Hospital La Fe Valencia Spain; ^8^ Pediatric Oncology Unit La Fe University Hospital Valencia Spain; ^9^ Department of Paediatrics St. Anna Children's Hospital and Children's Cancer Research Institute (CCRI) Medical University Vienna Austria

**Keywords:** 11q deletion, anti‐GD2 immunotherapy, combination immunotherapy, immune cell infiltration, miRNAs, neuroblastoma

## Abstract

High‐risk neuroblastoma (NB) patients with 11q deletion frequently undergo late but consecutive relapse cycles with fatal outcome. To date, no actionable targets to improve current multimodal treatment have been identified. We analyzed immune microenvironment and genetic profiles of high‐risk NB correlating with 11q immune status. We show in two independent cohorts that 11q‐deleted NB exhibits various immune inhibitory mechanisms, including increased CD4+ resting T cells and M2 macrophages, higher expression of programmed death‐ligand 1, interleukin‐10, transforming growth factor‐beta‐1, and indoleamine 2,3‐dioxygenase 1 (*P* < 0.05), and also higher chromosomal breakages (*P* ≤ 0.02) and hemizygosity of immunosuppressive miRNAs than MYCN‐amplified and other 11q‐nondeleted high‐risk NB. We also analyzed benefits of maintenance treatment in 83 high‐risk stage M NB patients focusing on 11q status, either with standard anti‐GD2 immunotherapy (*n* = 50) or previous retinoic acid‐based therapy alone (*n* = 33). Immunotherapy associated with higher EFS (50 vs. 30, *P* = 0.028) and OS (72 vs. 52, *P* = 0.047) at 3 years in the overall population. Despite benefits from standard anti‐GD2 immunotherapy in high‐risk NB patients, those with 11q deletion still face poor outcome. This NB subgroup displays intratumoral immune suppression profiles, revealing a potential therapeutic strategy with combination immunotherapy to circumvent this immune checkpoint blockade.

## Graphical abstract

High‐risk 11q‐deleted neuroblastomas display features of a higher immunosuppression microenvironment than other high‐risk neruoblastomas. Efficacy of current anti‐GD2 immunotherapy in 11q‐deleted neuroblastomas may be reduced by inhibition of effector cells by kynurenine production and tryptophan depletion by IDO1, polarized M2 macrophages, PD‐L1 expression, and IL‐10‐dependent Treg conversion from resting CD4+ T cells, providing a rationale for further combination immunotherapy studies.

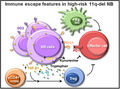

**Abbreviations**
11q‐del: 11q‐deleted
ADCC: antibody‐dependent cellular cytotoxicity
CDC: complement‐dependent cytotoxicity
COJEC: chemotherapeutic agents cisplatin, vincristine, carboplatin, etoposide, and cyclophosphamide
CTLA‐4: cytotoxic T lymphocyte antigen 4
EFS: event‐free survival
FISH: fluorescence in situ hybridization
HR: hazard ratio
ICI: immune checkpoint inhibitor
IDO1: indoleamine 2,3‐dioxygenase 1
IFN‐γ: interferon‐γ
IL‐10: interleukin 10
INRG: International Neuroblastoma Risk Group
miR: microRNA
MLPA: multiplex ligation‐dependent probe amplification
MMR: mismatch repair
MNA: *MYCN* amplification
MS: metastatic special stage
MSI: microsatellite instability
NB: neuroblastoma
NCA: numerical chromosome aberrations
NOS: nitric oxide synthase
OS: overall survival
PD‐1: programmed cell death protein 1
PD‐L1: programmed death‐ligand 1
SCA: segmental chromosome aberrations
TAM: tumor‐associated macrophages
Tfh: follicular helper T cells
TGF‐β: tumor growth factor‐β
TMB: tumor mutational burden
TME: tumor microenvironment
TNF‐α: tumor necrosis factor‐α
Treg: regulatory T cells

## 1 Introduction

Neuroblastoma is the most common extracranial solid tumor in childhood [1]. The OS for patients with low‐risk disease is 85–90%. In contrast, more than half of the children diagnosed with the high‐risk subtype will either not respond to current therapies or relapse after treatment, with a postrelapse OS less than 10–20% [2]. Biological factors associated with increased risk for disease progression include chromosomal alterations in 11q, 3p, 1p, and *MYCN* amplification (MNA) [3]. Among them, 11q and MNA are the most frequent (30% and 20%, respectively), and thus, they are considered as stratifying prognostic markers by the International Neuroblastoma Risk Group (INRG) staging system [4]. Interestingly, MNA inversely correlates with 11q deletion [3, 5]. However, *MYCN* is yet undruggable and the genetic basis for 11q deletion pathogenesis is unclear.

Genetic imbalance in 11q has been the focus of multiple studies (reviewed in [6]) aiming to understand the clinical implications and the etiology of this NB subtype. Most of the cases are diagnosed at an older age in the high‐risk group, displaying higher relapse probability and dismal prognosis [7, 8, 9, 10]. Despite the uncertain genetic etiology in 11q‐deleted NB, genomic instability is a hallmark of this tumor subset and represents a weakness that can be therapeutically exploited. In contrast to MNA subtype, a high frequency of chromosomal breaks is observed in these tumors, suggesting chromosomal instability [11]. These observations point out the plausible implication of DNA repair genes in 11q pathogenesis, either by haploinsufficiency or by inactivation of the remaining allele by mutation or epigenetic alterations [9, 12]. Thus, genomic instability would also explain the frequent tumor relapse and progression associated with 11q‐deleted NB patients, facilitating tumor cells to escape treatment.

Optimal treatment for minimal residual disease is crucial to prevent relapse. Maintenance antibody‐dependent cellular cytotoxicity treatment with anti‐GD2 immunotherapy has become the standard of care for patients with high‐risk NB. Targeting tumor cell surface with antibodies against ganglioside GD2 has been shown to eradicate tumor cells by both, induction of (ADCC), mediated mainly by NK cells, monocytes, neutrophils, and macrophages [13, 14] and by complement‐dependent cytotoxicity (CDC) [15, 16]. However, abreast of its significant toxicity, only a subset of high‐risk NB patients respond to it [17]. Thus, it is crucial to define the determinants driving the effectiveness and resistance to standard immunotherapy and consider novel strategies for unresponsive tumors. Patients with 11q‐deleted NB are less likely to respond to induction therapies [18]; however, their response to maintenance immunotherapy has not yet been defined. Currently, the degree of tumor‐infiltrating immune cells and tumor genomics are considered as determinants of immunotherapy response in other tumors [19]. Hence, the major objective of this study was to identify intratumoral biomarkers of antitumor immune response by integrating immune and genomic profiling of these tumors. These intratumoral biomarkers are of clinical relevance as they reveal therapeutic alternatives for nonresponding patients.

## 2 Materials and methods

### 2.1 Patients

We retrospectively evaluated benefits of immunotherapy during maintenance treatment by comparing outcome between high‐risk stage M NB patients treated with anti‐GD2 immunotherapy plus retinoic acid (*n* = 50) and patients treated with retinoic acid‐based therapy alone (*n* = 33) (Table 1). All patients were enrolled at HR‐NBL/SIOPEN trial between 2007 and 2015. Immunotherapy arm was activated in Spain in 2010. Hence, patients receiving retinoic acid‐based therapy alone were treated before 2010 in accordance to HR‐NBL/SIOPEN trial. All patients included in the study had completed intensive induction therapy (rapid COJEC or modified N7) with or without two additional cycles of TVD (topotecan–vincristine–doxorubicin), followed by surgery, myeloablative therapy, and radiotherapy [20]. Time between the start of induction chemotherapy and the start of high‐dose chemotherapy followed by peripheral blood stem cell rescue was less than 9 months. More details in patient eligibility and treatment were previously described [20].

**Table 1:** Clinical characteristics of the Spanish high‐risk NB (M stage) cohort enrolled at HR‐NBL/SIOPEN trial.

Characteristics	*n* (%)		
	Immunotherapy	RA alone	Total
Treatment group	50 (60.2)	33 (39.8)	83 (100)
Sex			
Male	30 (60)	17 (51.5)	47 (56.6)
Female	20 (40)	16 (48.5)	36 (43.4)
INRG stage			
M	50 (100)	33 (100)	83 (100)
MYCN status			
Amplified	14 (28)	11 (33.3)	25 (30.1)
Not amplified	36 (72)	22 (66.7)	58 (69.9)
11q status			
Deleted	24 (48)	11 (33.3)	35 (42.2)
Nondeleted	26 (52)	22 (66.7)	48 (57.8)
Relapse			
Yes	31 (62)	25 (75.8)	56 (67.5)
No	19 (38)	8 (24.2)	27 (32.5)
Patient status			
Dead	25 (50)	24 (72.7)	49 (59)
Alive	25 (50)	9 (27.3)	34 (41)

Patients were staged according to the INRG classification system [4]. Biological studies included status of MYCN (studied by FISH) and 11q (studied by MLPA from 2008–2012 and by CytoScanHD arrays from 2013–2016), according to ENQUA guidelines. Clinical and follow‐up data were obtained from Spanish neuroblastoma studies database. The study was conducted in accordance with the reporting recommendations for tumor marker prognostic studies (REMARK); the Declaration of Helsinki and La Fe Research Ethics Committee approved this project. Parents or legal guardians signed an informed consent statement for sample and data management.

### 2.2 Single nucleotide polymorphism arrays

High‐resolution SNP arrays analysis was performed in 41 metastatic NB patients. Out of the 41, 29 were classified as stage M and 12 as stage MS (Metastatic Special). We included MS cases to identify SCA differences between both metastatic subgroups. Whole‐genome copy number variations were analyzed by SNP arrays (CytoScan HD, Thermo Fisher Scientific, Inc., Waltham, MA, USA) as previously described [9]. Briefly, isolated DNA from fresh tumor was fragmented by Nsp I digestion and further ligated to adaptor followed by PCR amplification. The PCR product was hybridized using Affymetrix CytoScan HD Array Gene Chip and processed with the Fluidic Station ( Thermo Fisher Scientific, Inc.). SNP array results were analyzed with Chromosome Analysis Suite software (chas v3.1; Thermo Fisher Scientific, Inc.). The annotation version used by the chas software is based on the February 2009 human reference sequence GRCh37 (hg19).

### 2.3 Tumor‐infiltrating immune cells analysis

RNA expression profiles of 55 high‐risk primary NB derived from the Westermann cohort [21] were extracted from R2 Genomics Analysis and Visualization Platform (https://r2.amc.nl). The dataset (GSE73517) contained 18 patients with 11q‐deletion, 27 patients with MNA, and five patients with neither of the two alterations. We identified five patients with both alterations but they were excluded from this study. Tumor‐infiltrating immune cells in each tumor subgroup were assessed using CIBERSORTx (https://cibersortx.stanford.edu/) [22]. Gene expression data from high‐risk NB patients were input as a mixture file. LM22 signature matrix was used to distinguish 22 immune cell types, including naïve B cells, memory B cells, plasma cells, CD8+ T cells, naïve CD4+ T cells, resting CD4+ memory T cells, activated CD4+ memory T cells, follicular helper T cells (Tfh), Tregs, gamma delta T cells (γδ T cells), resting NK cells, activated NK cells, monocytes, M0 macrophages, M1 macrophages, M2 macrophages, resting dendritic cells (DCs), activated DCs, resting mast cells, activated mast cells, eosinophils, and neutrophils. Bulk‐mode batch correction was applied to remove variances between platforms. Expression data were quantile normalized. Permutations were set to 1000, and the algorithm was run in absolute mode. Samples with a deconvolution *P*‐value < 0.05 were accepted. Scores represent the absolute proportion of each cell type in the mixture. For duplicated gene symbols, the one with the highest mean across samples was selected. To validate the results, we used the data from the Therapeutically Applicable Research to Generate Effective Treatments (TARGET) project (GSE131189). Of the total cohort, 215 patients belonged to the high‐risk NB subgroup and had both expression and CNV data [23]. The validation cohort was composed of 95 patients with 11q‐deletion, 56 patients with MNA, 60 patients with neither of the two alterations, and 4 with both alterations that were excluded from this study.

### 2.4 MiRNA analysis

11q allocated miRNAs‐targeted genes were obtained using TargetScan (http://www.targetscan.org/) [24]. miRNA sequences and annotation data (MIID or precursor miRNA and location) were acquired from the miRbase database (http://mirbase.org/) [25]. We searched for the presence of conserved 8mer, 7mer, and 6mer sites that match the seed region of each miRNA [26]. Predictions with only poorly conserved sites were excluded. Sites with mismatches in the seed region that are compensated by conserved 3′ pairing [27] and centered sites [28] were included. Predictions were ranked based on the predicted efficacy of targeting as calculated using cumulative weighted context++ scores of the sites [24]. Threshold predictive value used in databases was 0.9 or higher to insure highest prediction in the interaction miRNA‐mRNA. Algorithm used to identify targeted genes is based on collected data in TargetScan (conserved site context scores, version 7.1), miRDB (release 5.0) and validated information from miRTarBase (version 7.0). The expression of predicted genes affected by miRNAs was further analyzed on the Westermann dataset and defined as intratumoral affected genes. cytoscape 3.7.1. was used to integrate and visualize the results from the GSEA [29]. r was used for data collection and analysis.

### 2.5 Statistical analysis

The primary endpoints were 3‐ and 5‐year EFS and OS. EFS was defined as the time after postconsolidation therapy to the first relapse or progression, death or second neoplasm, or to last follow‐up. OS was calculated from the time of postconsolidation therapy to death from any cause or to last follow‐up. Univariate OS and EFS analyses were performed using Cox proportional hazards regression. SCA profiles between subgroups were tested using one‐way analysis of variance (one‐way ANOVA) with SPSS v21 (IBM Corp., Armonk, NY, USA). Expression levels were converted to log2 values for statistical analysis. Three‐way ANOVA was used to assess differences in gene expression between patients with 11q‐deletion, MNA, and patients without 11q or MNA (others). Samples with a deconvolution *P*‐value < 0.05 were included in the analysis. Ordinal regression model was used to assess the difference between the three subgroups based on immune cell population proportions. Statistical analyses were performed using r version 4. Stacked bar chart and heatmap table with absolute immune scores were generated with CIBERSORTx. Boxplots were generated using graphpad prism v9 (graphpad software inc., san diego, ca, usa). *P* values < 0.05 were considered statistically significant.

## 3 Results

### 3.1 Immune landscape of high‐risk NB

**Fig. 1:**
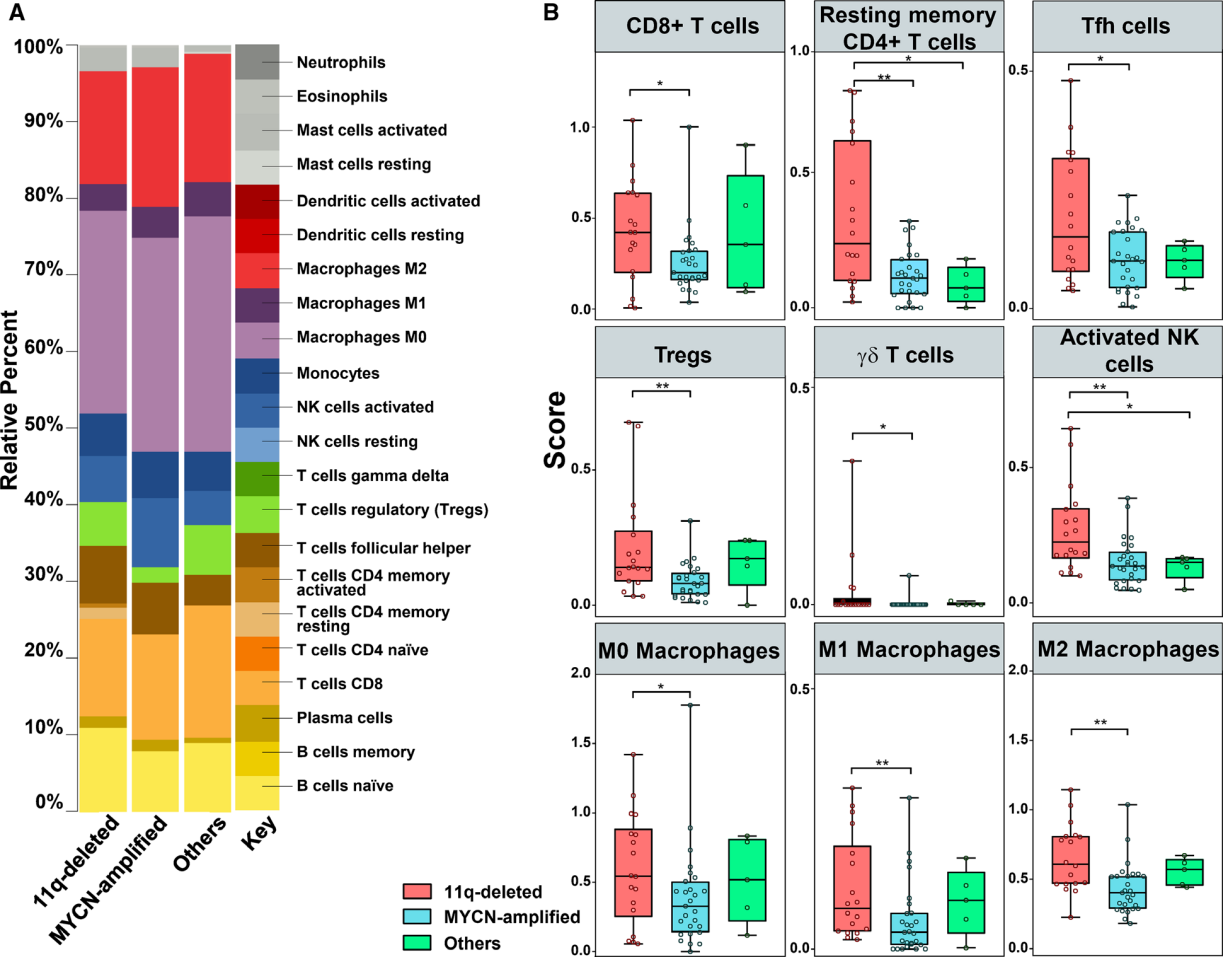
Immune profiling characteristics in 11q‐deleted NB tumors. (A) CIBERSORTx absolute inferred composition of immune cell subsets in high‐risk NB patients. Data obtained from the Westermann cohort at R2 genomics analysis and visualization platform (https://r2.amc.nl). (B) Boxplot of tumor‐infiltrating immune populations of high‐risk NB with 11q deletion (*n* = 18; red boxes), MYCN‐amplified (*n* = 27; blue boxes), and other high‐risk NB (*n* = 5; green boxes), as indicated. Ordinal regression model was used to assess the difference between the three subgroups based on immune cell population proportions. *P ≤ 0.05; **P < 0.01. Five high‐risk NB with both MNA and 11q deletion was excluded.

**Fig. 2:**
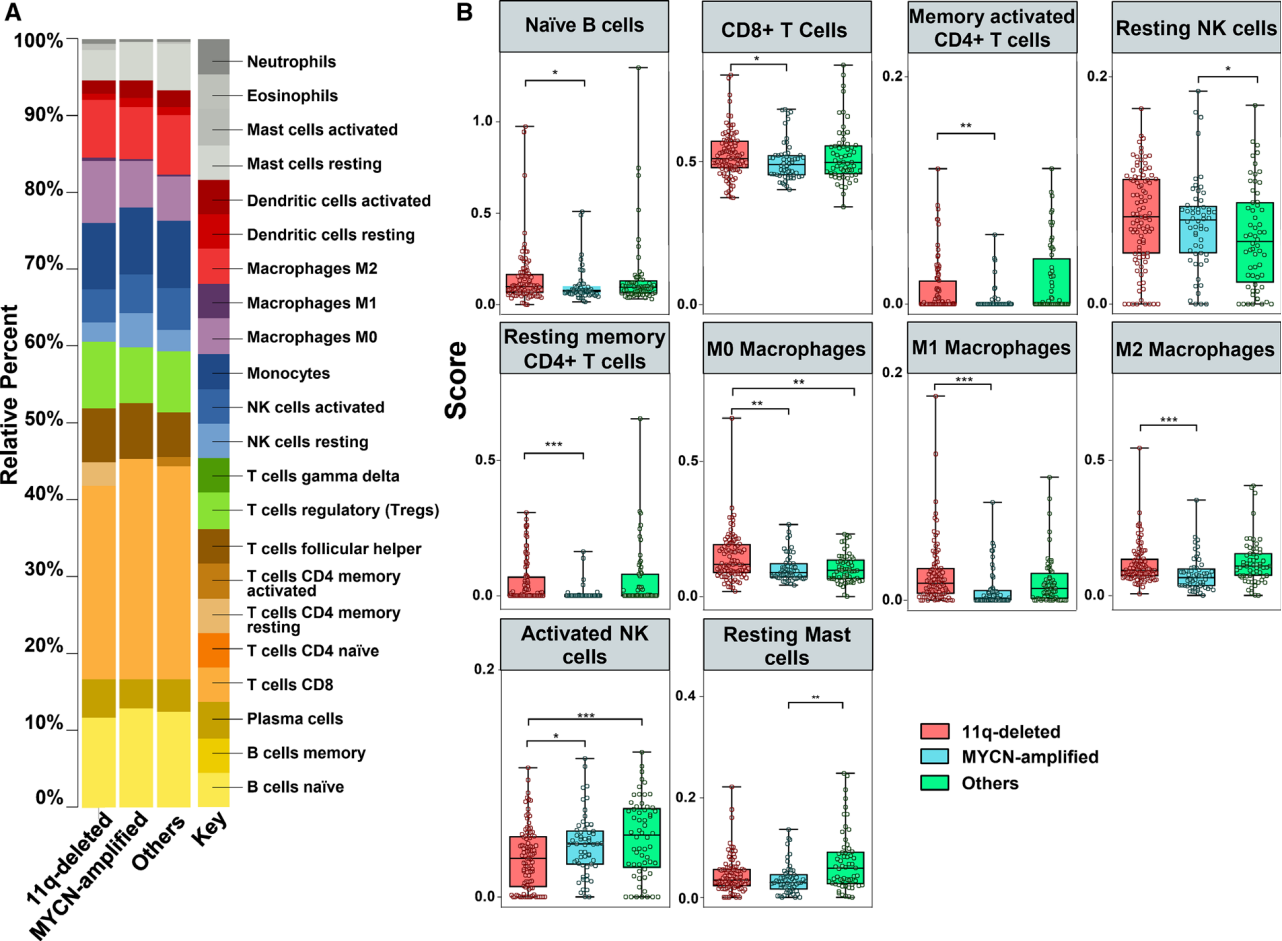
Validation cohort of the immune profiling characteristics in 11q‐deleted NB tumors. (A) CIBERSORTx absolute inferred composition of immune cell subsets in high‐risk NB patients. Data obtained from TARGET cohort at (https://ocg.cancer.gov/programs/target/projects/neuroblastoma). (B) Boxplot of tumor‐infiltrating immune populations of high‐risk NB with 11q‐deletion (*n* = 95; red boxes), MYCN‐amplified (*n* = 56; blue boxes), and other high‐risk NB (*n* = 60; green boxes), as indicated. Ordinal regression model was used to assess the difference between the three subgroups based on immune cell population proportions. *P ≤ 0.05; **P < 0.01. Four high‐risk NBs with both MNA and 11q deletion were not considered.

In order to study tumor‐infiltrating immune cells in high‐risk NB, we used CIBERSORTx for deconvolution of gene expression data. We applied an ordinal regression model to analyze the differential distribution of immune‐infiltrating cell subsets in the tumor microenvironment (TME). To determine the independent immune profiles of 11q‐deleted and MYCN‐amplified tumor subtypes, patients were divided into three subgroups: (a) 11q‐deleted (*n* = 18), (b) MYCN‐amplified (*n* = 27), and (c) 11q normal without MNA (*n* = 5), here referred as others. Patients with both 11q deletion and MNA (*n* = 5) were discarded as the independent effect of both alterations could not be assessed in these cases. Heatmap with absolute immune fraction scores can be found in Table S1. CIBERSORTx inferred a differential distribution of immune cell subsets between NB subgroups (Fig. 1A). Analysis of the ordinal regression model showed that the immune landscape between high‐risk NB subgroups displays significant differences (Table 2; Fig. 1B). 11q‐deleted NB displayed higher absolute proportion (score) of CD8+ T cells, Tregs, Tfh cells, γδ T cells, M0, M1, and M2 macrophages compared to MYCN‐amplified NB and higher resting CD4+ memory T cells and activated NK cells compared to MYCN‐amplified NB and others (*P* < 0.05) (Fig. 1B). A second cohort (TARGET) was used to validate the immune‐infiltrating cell subsets. Heatmap with absolute immune fraction scores is detailed in Table S2. Higher absolute proportion of CD8+ T cells, resting CD4+ memory T cells, M0, M1, and M2 macrophages were also found in 11q‐deleted NB compared to MYCN‐amplified NB (Table 3 and Fig. 2). Activated NK cells were significantly lower in 11q‐deleted NB compared to the other high‐risk NB subgroups (Tables 2 and 3). Of note, γδ T cells population was almost absent in both subgroups (Tables S1 and S2).

**Table 2:** Ordinal regression analysis of differential tumor‐infiltrating immune populations of Westermann high‐risk NB cohort.

Immune cell population	11q‐deleted vs.	Odds ratio	Std. error	*z* Value	*P* value
Naïve B cells	MYCN‐amplified	0.492	0.573	−1.238	0.216
	Others	0.659	0.885	−0.472	0.637
Memory B cells	MYCN‐amplified	0.441	0.569	−1.440	0.15
	Others	0.399	0.979	−0.939	0.348
Plasma cells	MYCN‐amplified	0.490	0.540	−1.325	0.185
	Others	0.668	0.843	−0.479	0.632
CD8+ T cells	MYCN‐amplified	0.233	0.585	−2.486	0.0129*
	Others	0.557	1.008	−0.581	0.5613
Naïve CD4+ T cells	MYCN‐amplified	0.767	NA	NA	NA
	Others	5.637E‐09	NA	NA	NA
Resting CD4+ memory T cells	MYCN‐amplified	0.171	0.595	−2.963	0.003**
	Others	0.101	0.912	−2.510	0.012*
Activated CD4+ memory T cells	MYCN‐amplified	1.130	0.665	0.184	0.854
	Others	2.798	0.927	1.111	0.266
Follicular helper T cells	MYCN‐amplified	0.283	0.578	−2.184	0.029*
	Others	0.318	0.833	−1.377	0.168
Tregs	MYCN‐amplified	0.190	0.578	−2.883	0.004**
	Others	1.184	0.880	0.193	0.847
γδ T cells	MYCN‐amplified	0.100	1.145	−2.009	0.045*
	Others	0.564	1.215	−0.472	0.637
Resting NK cells	MYCN‐amplified	Underrepresented	NA	NA	NA
	Others	model do not converge	NA	NA	NA
Activated NK cells	MYCN‐amplified	0.157	0.593	−3.127	0.002**
	Others	0.146	0.875	−2.200	0.028*
Monocytes	MYCN‐amplified	0.530	0.529	−1.201	0.230
	Others	0.436	0.971	−0.855	0.392
M0 macrophages	MYCN‐amplified	0.308	0.569	−2.071	0.0384*
	Others	0.727	0.866	−0.368	0.713
M1 macrophages	MYCN‐amplified	0.229	0.556	−2.649	0.008**
	Others	0.851	0.846	−0.191	0.84869
M2 macrophages	MYCN‐amplified	0.129	0.602	−3.405	0.000***
	Others	0.585	0.815	−0.658	0.510
Resting dendritic cells	MYCN‐amplified	0.588	0.629	−0.846	0.398
	Others	0.353	1.202	−0.867	0.386
Activated dendritic cells	MYCN‐amplified	Underrepresented	NA	NA	NA
	Others	model do not converge	NA	NA	NA
Resting mast cells	MYCN‐amplified	0.578	0.630	−0.875	0.382
	Others	3.090	0.893	1.263	0.207
Activated mast cells	MYCN‐amplified	1.246	0.543	0.405	0.686
	Others	0.237	0.965	−1.491	0.136
Eosinophils	MYCN‐amplified	0.772	0.555	−0.467	0.64
	Others	0.607	0.871	−0.573	0.567
Neutrophils	MYCN‐amplified	3.203	NA	NA	NA
	Others	2.261E‐08	NA	NA	NA

Signif. codes: ***0.001; **0.01; *0.05.

**Table 3:** Ordinal regression analysis of differential tumor‐infiltrating immune populations of TARGET high‐risk NB cohort.

Immune cell population	11q‐deleted vs.	Odds ratio	Std. error	*z* Value	*P* value
Naïve B cells	MYCN‐amplified	0.494	0.293	−2.403	0.016*
	Others	0.836	0.288	−0.624	0.532
Memory B cells	MYCN‐amplified	0.741	0.340	−0.881	0.379
	Others	1.060	0.320	0.181	0.856
Plasma cells	MYCN‐amplified	0.735	0.287	−1.072	0.284
	Others	1.034	0.293	0.113	0.910
CD8+ T cells	MYCN‐amplified	0.550	0.290	−2.406	0.040*
	Others	0.757	0.292	−0.955	0.340
Naïve CD4+ T cells	MYCN‐amplified	0.560	0.387	−1.499	0.134
	Others	0.524	0.397	−1.629	0.103
Resting CD4+ memory T cells	MYCN‐amplified	0.156	0.427	−4.353	1.340e‐05***
	Others	0.847	0.311	−0.535	0.592
Activated CD4+ memory T cells	MYCN‐amplified	0.286	0.398	−3.145	0.002**
	Others	1.370	0.316	0.997	0.319
Follicular helper T cells	MYCN‐amplified	0.714	0.296	−1.138	0.255
	Others	0.731	0.285	−1.099	0.272
Tregs	MYCN‐amplified	1.077	0.283	0.262	0.793
	Others	0.665	0.296	−1.378	0.168
γδ T cells	MYCN‐amplified	0.000	NA	NA	NA
	Others	1.637	NA	NA	NA
Resting NK cells	MYCN‐amplified	0.746	0.286	−1.026	0.305
	Others	0.466	0.297	−2.571	0.010*
Activated NK cells	MYCN‐amplified	1.983	0.286	2.397	0.017*
	Others	2.851	0.305	3.440	0.000***
Monocytes	MYCN‐amplified	0.567	0.290	−1.956	0.051
	Others	1.119	0.292	0.386	0.700
M0 macrophages	MYCN‐amplified	0.402	0.293	−3.119	0.002**
	Others	0.384	0.296	−3.236	0.001**
M1 macrophages	MYCN‐amplified	0.187	0.317	−5.291	1.220e‐07***
	Others	0.590	0.286	−1.842	0.065
M2 macrophages	MYCN‐amplified	0.297	0.308	−3.949	7.850e‐05***
	Others	1.195	0.287	0.620	0.535
Resting dendritic cells	MYCN‐amplified	0.962	0.311	−0.125	0.900
	Others	1.521	0.303	1.383	0.167
Activated dendritic cells	MYCN‐amplified	0.777	0.290	−0.870	0.384
	Others	1.064	0.300	0.208	0.835
Resting mast cells	MYCN‐amplified	0.659	0.288	−1.447	0.148
	Others	2.287	0.298	2.772	0.006**
Activated mast cells	MYCN‐amplified	1.822	0.365	1.644	0.100
	Others	0.876	0.395	−0.335	0.738
Eosinophils	MYCN‐amplified	2.060	0.583	1.240	0.215
	Others	1.326	0.629	0.448	0.654
Neutrophils	MYCN‐amplified	1.847	0.331	1.853	0.064
	Others	1.275	0.337	0.723	0.470

Signif. codes: ***0.001; **0.01; *0.05.

### 3.2 High‐risk neuroblastoma response to anti‐GD2 immunotherapy

**Fig. 3:**
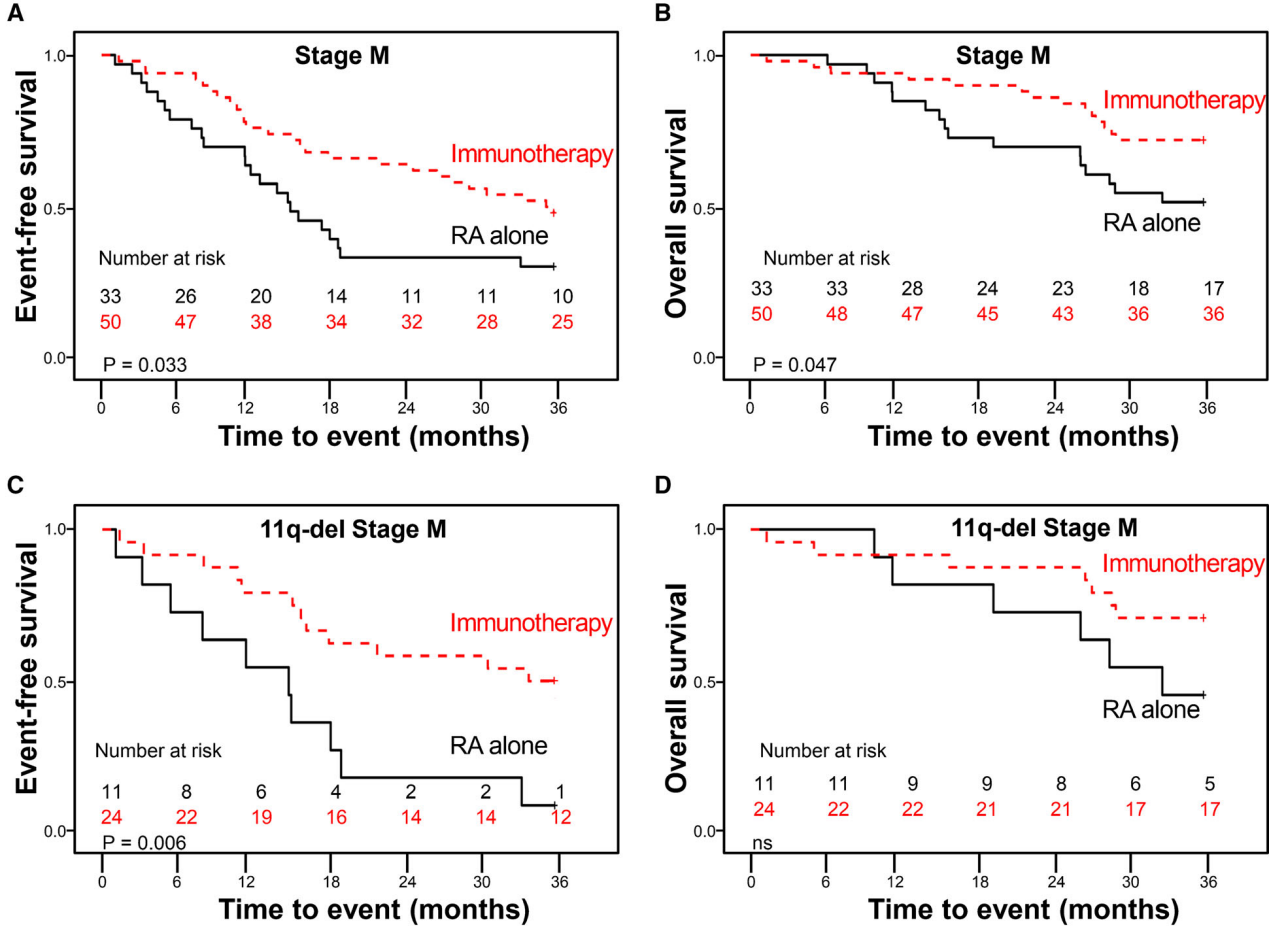
Kaplan–Meier curves of 3‐year EFS for 11q‐deleted NB patients according to treatment with anti‐GD2 immunotherapy (dashed line) or RA alone treatment (straight line). Censored cases are denoted as crosses along the plots. Number of patients still at risk are indicated in each plot after removing those censored patients from the denominator. Log‐rank *P* values were used to compare curves between subgroups. (A) EFS and (B) OS in 83 high‐risk stage M neuroblastoma patients treated with immunotherapy or conventional maintenance treatment. (C) EFS and (D) OS in the subgroup of 11q‐deleted stage M patients (*n* = 36); NS, no significance *P* > 0.05.

Immune profile in 11q‐deleted NB depicts some potential immune checkpoints previously reported to be involved in immune scape and immune tolerance to immunotherapy (anti‐HER1/2 immunotherapy in breast tumors) [30]. Following this line of reasoning, we evaluated outcome in a cohort of 83 high‐risk NB patients (M stage) subjected to maintenance treatment, focusing on 11q subgroup (Table 4). Immunotherapy significantly improved survival rates (EFS and OS) in the overall cohort (Table 4; Fig. 3A,B). However, immunotherapy in the 11q‐deleted NB subgroup only significantly improved EFS but not OS (Table 4; Fig. 3C,D).

**Table 4:** Effect of treatment on survival in high‐risk NB (M stage) patients. RA: 13‐cis retinoic acid (isotretinoin). HR from log‐rank tests. CI, confidence interval; HR, hazard ratio.

Subgroup	Treatment group	3‐year outcome						
		*N*	EFS (95% CI)	HR (95% CI)	*P*	OS (95% CI)	HR (95% CI)	*P*
All patients	Immunotherapy	50	50 (38–66)	0.53 (0.30‐ 0.94)	0.028	72 (61–86)	0.49 (0.24–1)	0.047
	RA alone	33	30 (18–51)			52 (37–72)		
11q‐deleted	Immunotherapy	24	50 (34–75)	0.32 (0.14–0.76)	0.006	71 (55–92)	0.46 (0.15–1.37)	0.152
	RA alone	10	9 (1–59)			46 (24–87)		

Beyond the known late relapse frequency in 11q‐deleted NB, our data suggest that their relapse may be delayed by anti‐GD2 immunotherapy, but outcome still remains poor. Identifying specific biological and molecular peculiarities in 11q‐deleted NB subgroup may provide additional targets to enhance the current treatment. One plausible explanation is that perhaps some immune checkpoints in this subtype may be impeded.

**Fig. 4:**
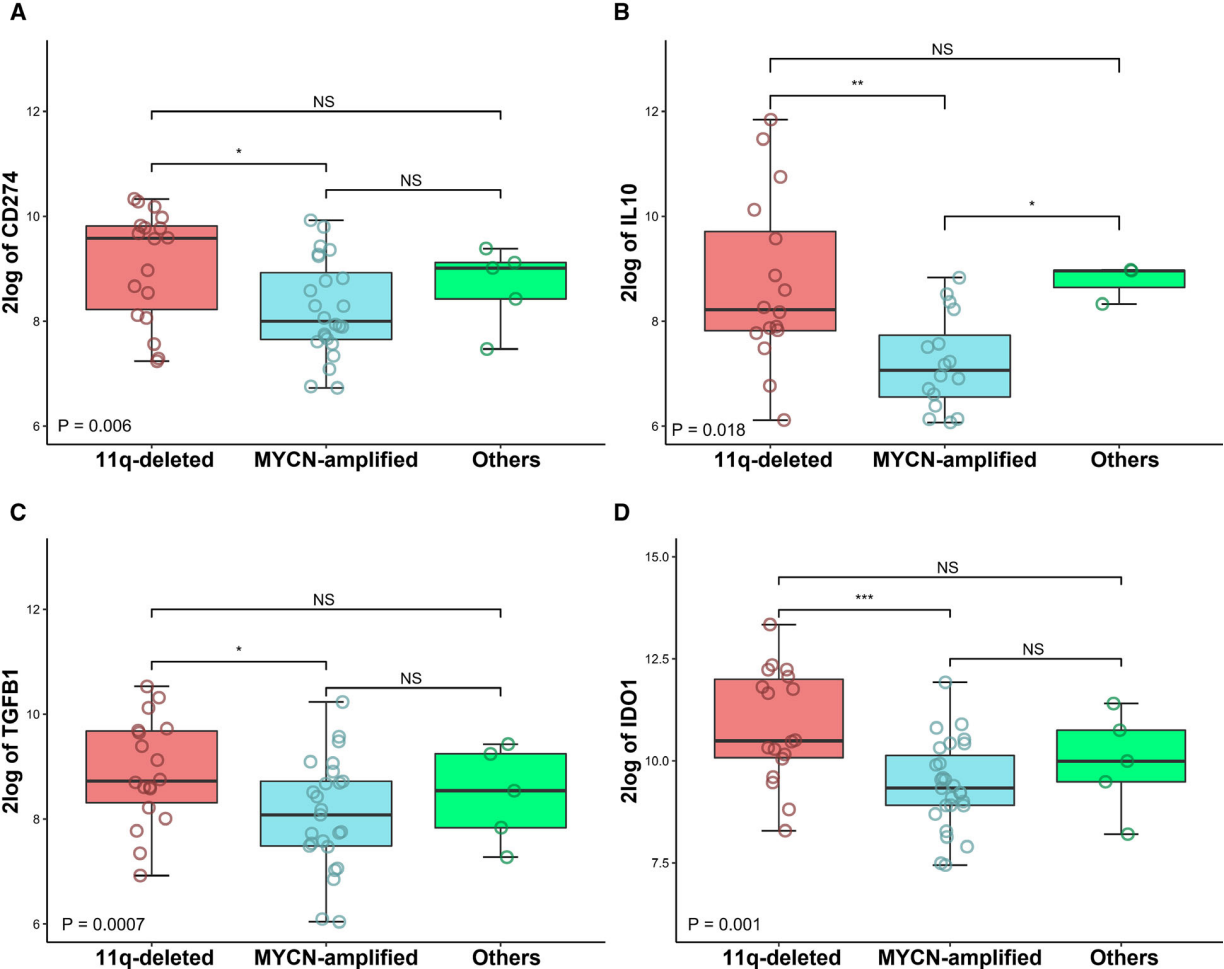
Expression of immunosuppressive genes in 11q‐deleted high‐risk NB. Differential expression of (A) CD274 (PD‐L1), (B) IL‐10, (C) TGFB1 (TGF‐β1), (D) IDO1, in 11q‐deleted high‐risk (*n* = 18; red boxes), MYCN‐amplified (*n* = 27; blue boxes), and other high‐risk NB (*n* = 5; green boxes). Three‐way analysis of variance (ANOVA) Boxplots were generated with r from RNA expression profiles of 50 primary high‐risk NB derived from Westermann cohort at R2 genomics analysis and visualization platform (https://r2.amc.nl). Global *P* value is shown. *P ≤ 0.05; **P < 0.01; ***P < 0.001; NS: no significance. Five high‐risk NBs with both MNA and 11q deletion were excluded from study.

**Fig. 5:**
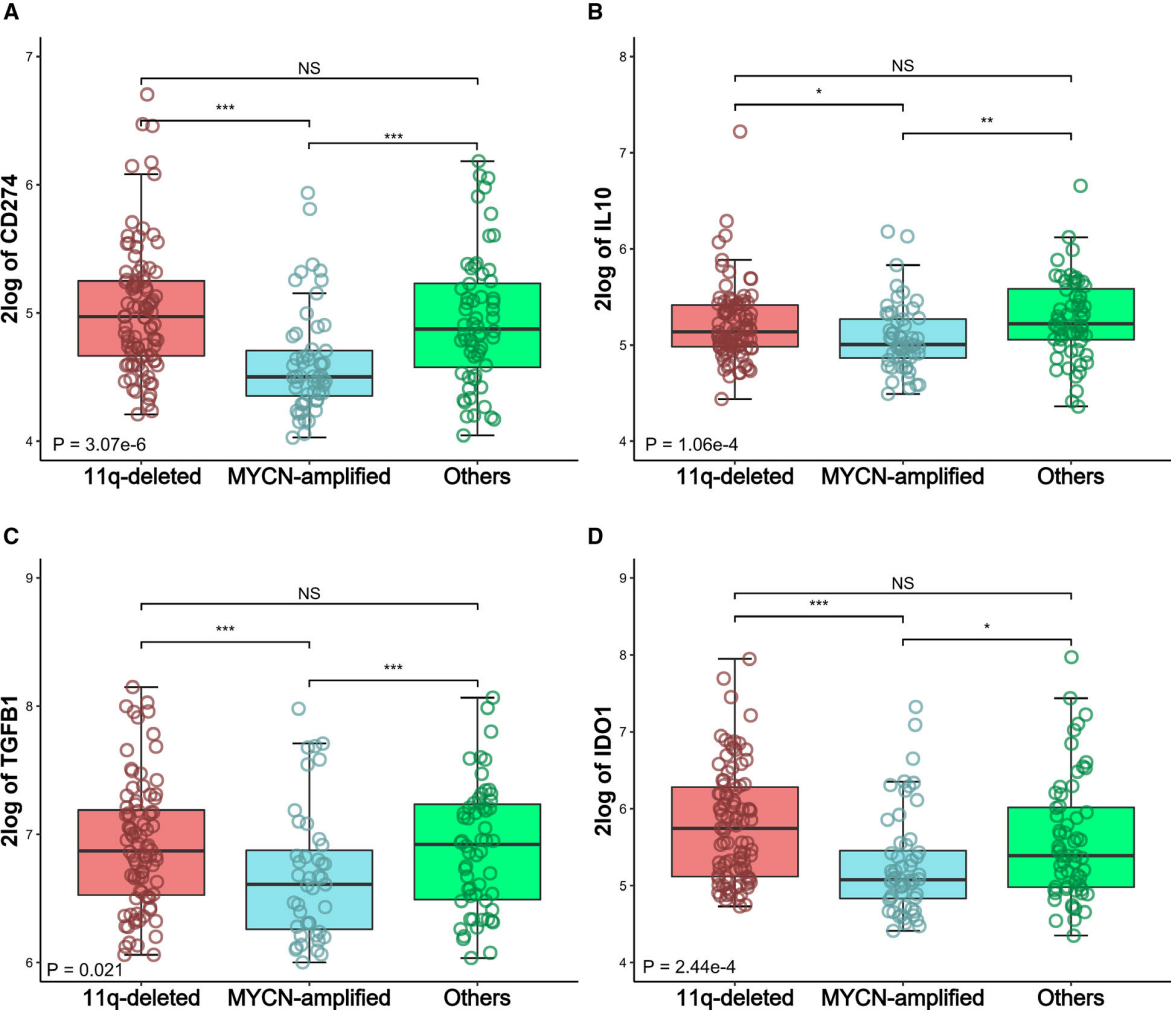
Validation cohort of the expression of immunosuppressive genes in 11q‐deleted high‐risk NB. Differential expression of (A) CD274 (PD‐L1), (B) IL‐10, (C) TGFB1 (TGF‐β1), (D) IDO1, in 11q‐deleted high‐risk NB (*n* = 95; red boxes), MYCN‐amplified (*n* = 56; blue boxes), and other high‐risk NB (*n* = 60; green boxes). Three‐way analysis of variance (ANOVA) boxplots were generated with r from RNA expression profiles of 211 primary high‐risk NB derived from TARGET cohort. Global *P* value is shown. *P ≤ 0.05; **P < 0.01; ***P < 0.001; NS, no significance. Five high‐risk NBs with both MNA and 11q deletion were not considered.

We next assessed the differential expression of immune checkpoint genes in 11q‐deleted, MYCN‐amplified, and 11q normal without MNA. Interestingly, 11q‐deleted NB showed significantly higher levels of the immune checkpoint PD‐L1 (Fig. 4A), as well as the immunosuppressive molecules IL‐10 (Fig. 4B), TGF‐β1 (Fig. 4C), and IDO1 (Fig. 4D), compared to MYCN‐amplified NB. These results were further validated in the TARGET dataset (Fig. 5), supporting the immunosuppression expression profile in 11q‐deleted NB tumors.

### 3.3 Whole‐genome copy number analysis

**Fig. 6:**
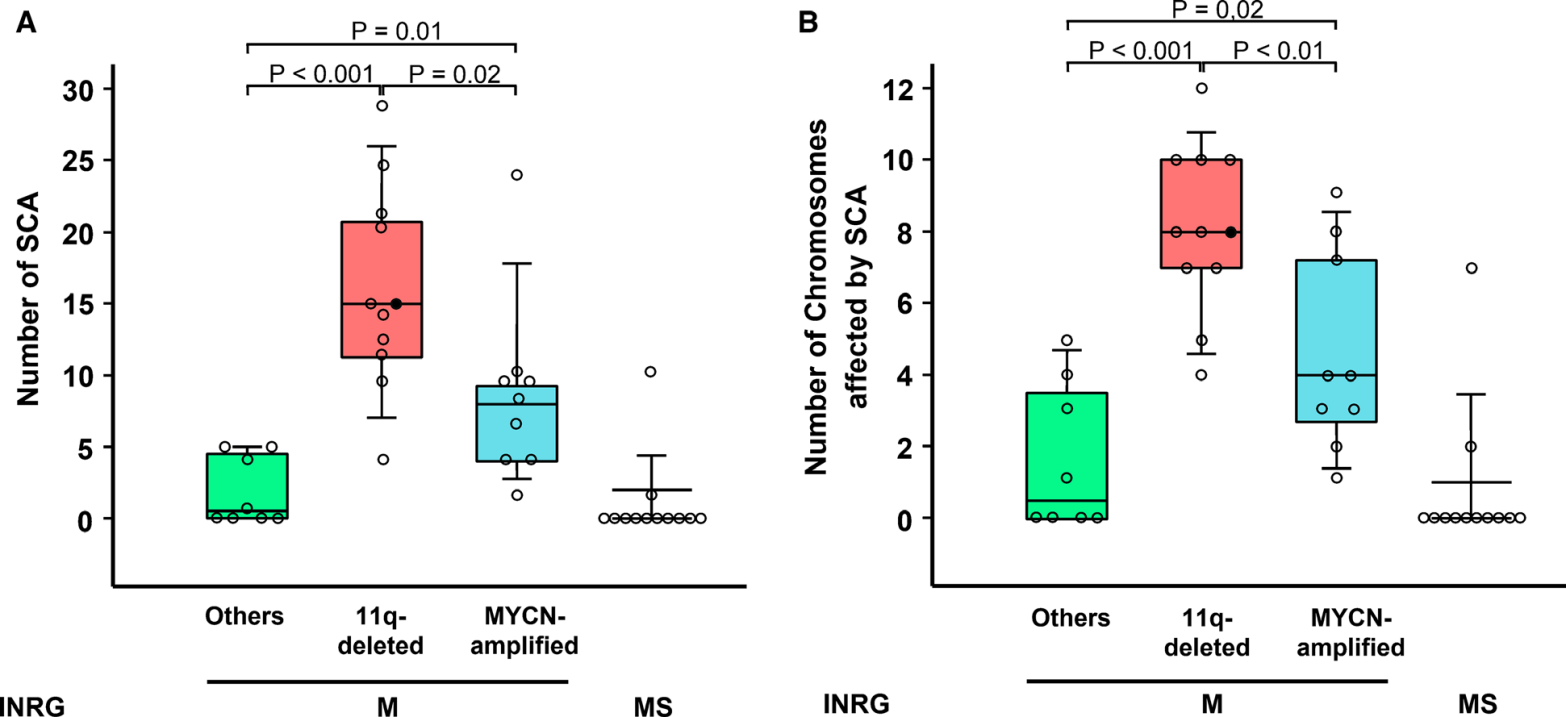
Segmental Chromosomal Aberrations (SCA) in stage M neuroblastomas identifies 11q‐deleted subgroup with higher SCA incidence. (A) Box plot representation of 41 stage M/MS NB tumors (median age at diagnosis 36.8 months, range 0.3–312.8 months, MNA 10, 11q deletion 12, others 8, stage MS 12) vs. total SCA determined for each case based on the molecular karyotype analysis with high‐resolution SNP arrays performed as previously described [9]. MS are not high‐risk group and have a more favorable outcome than high‐risk group, but were included in this SCA study to further illustrate their significant SCA differences, independently of their metastatic condition, in comparison with the high‐risk MNA and 11qdel subgroups. One case containing both MNA and 11q deletion is indicated with a closed circle. (B) Box plot representation for M/MS stage NB tumors as in (A) vs. the number of chromosomes affected in each case. Total number of chromosomes affected by SCA was *n* = 13 for (–); *n* = 89 for 11q‐del; *n* = 41 for MNA and *n* = 9 for MS.

While there is a paucity of recurrent somatic mutations among NB tumors, they frequently exhibit numerical chromosome aberrations (NCAs) and/or SCAs. We performed SNP array analysis on 41 high‐risk NB and confirmed a significantly higher total SCA number in 11q‐deleted subgroup than other high‐risk subgroups (Fig. 6A). Only one case contained both MNA and 11q deletion (indicated with a closed circle in Fig. 6A). In addition, we also studied MS patients without MNA or 11q deletion to demonstrate that this group had the lowest average number of SCAs among metastatic NB patients (Fig. 6A). Only two MS cases presented SCAs. Similarly, 11q‐deleted subgroup had significantly more SCA‐affected chromosomes than other subgroups, including MNA, further supporting previous report (Fig. 6B) [11].

### 3.4 MiRNA target enrichment analysis

Recently, miRNAs have been placed under the spotlight as responsible for aggressive disease in NB, either through the activation of effector cells or through the down‐regulation of regulatory cells [31]. Six miRNAs are located within 11q smallest region of overlapping deletion (SRO) (11q22.3‐11q23.3) [9]: *miR‑4491*, *miR‐34b*, *miR‐34c*, *miR‐4301*, *miR‐6716*, and *miR‐4492*. MiRNA details are described in Table S3. To uncover the multiple regulatory interactions between 11q miRNAs and their functional target genes, we attempted to predict miRNA‐mRNA regulatory modules in the constructed regulatory network. A miRNA‐mRNA regulatory module consists of a set of miRNAs and a set of their targets, in which the miRNAs coordinately regulate their targets. By combining known and bioinformatics based predicted targeted genes, we generated a network map of the biological pathways affected by the 11q miRNAs set (Table S4, Table 2). Response to cytokine (GO:0034097), negative regulation of immune system process (GO:0002683), and protein dephosphorylation (GO:0006470) constitute the three most significant biological processes affected by miRNAs localized in 11q SRO (Table S4, each GO term is indicated in single tabs).

**Fig. 7:**
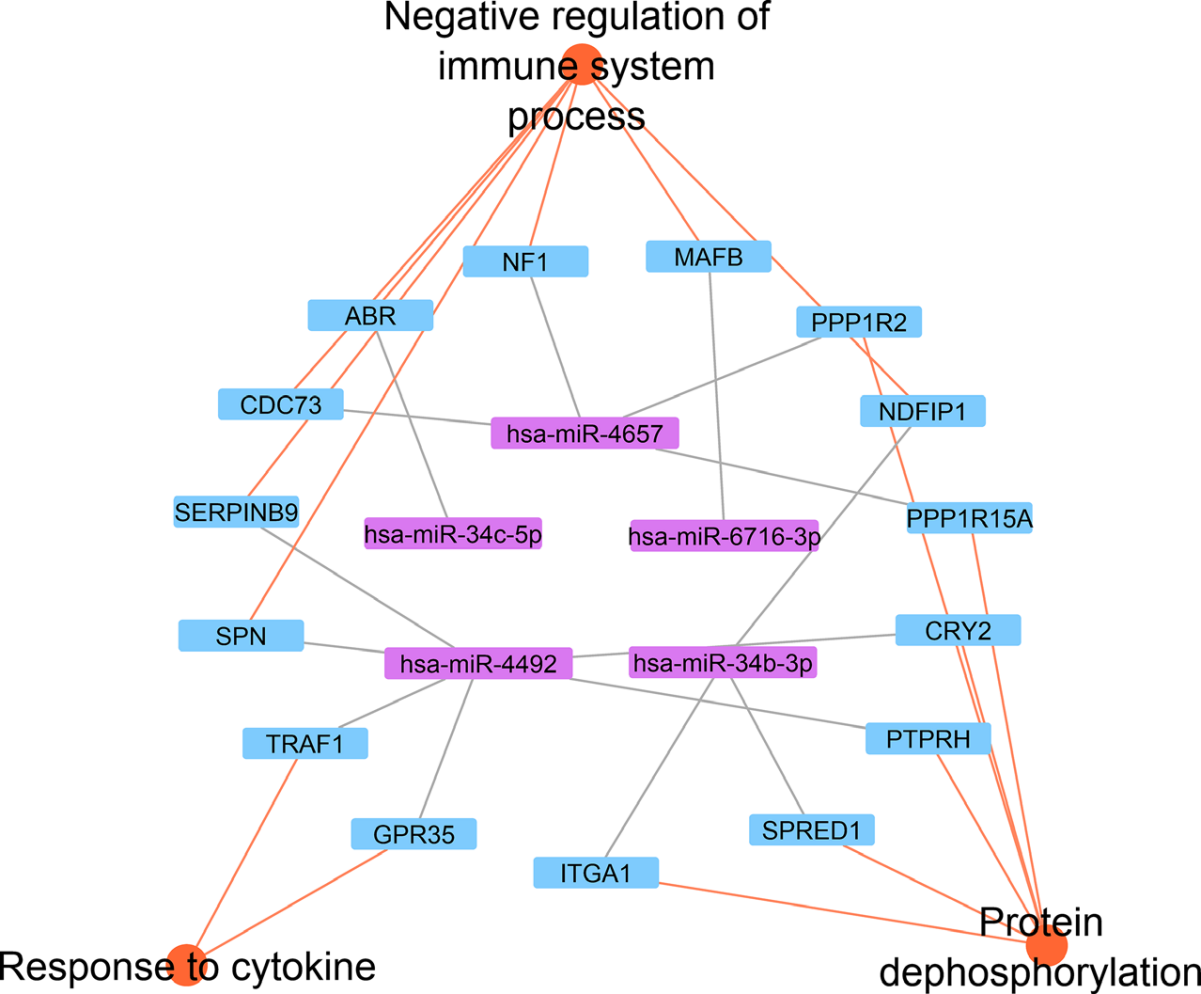
Intratumoral regulatory network by 11q‐deleted miRNAs set in neuroblastomas. Purple nodes in the inner circle constitute the miRNAs localized in 11q‐deleted SRO; blue nodes in the outer circle represent the known/predicted targeted genes that also showed differential expression in high‐risk 11q‐deleted NB. Orange nodes represent the indicated enriched biological processes, as described in Table S4. Lines between nodes represent interactions connecting miRNAs with genes and biological processes.

To further support our hypothesis of epigenetic regulation by 11q miRNAs, we analyzed the differential expression of these genes in the Westermann cohort using R2 genomics analysis and visualization platform (https://r2.amc.nl). Notably, 31% of the predicted genes displayed a differential intratumoral expression in high‐risk NB with 11q deletion (Fig. 7 and Table S5). These results strengthen our hypothesis and provide a new mechanism for the epigenetic regulation of immune response in 11q‐deleted NB.

## 4 Discussion

Neuroblastoma patients with 11q deletion undergo consecutive relapses with poor outcome, closely comparable to those with MNA [9]. The incorporation of anti‐GD2 immunotherapy into the maintenance treatment regimen has improved the outcome of high‐risk NB. However, there is a variable response to the therapy [20]. In this study, we identify immunosuppressive profile signatures supporting that combination immunotherapy may be more efficient in the treatment of high‐risk 11q‐deleted NB. To improve long‐term survival avoiding unnecessary sequela after treatment, novel therapies are required for 11q‐deleted NB patients.

To date, there are no definitive biomarkers that predict patient response to immunotherapy. The emerging picture is that a combination of tumor genetics and its immune environment determine antitumor immunity [19]. We used CIBERSORTx to characterize differential immune cell infiltration among high‐risk NB patients. Recent evidence has linked MYCN‐amplified NB with immunosuppression [32]. However, all risk subgroups but not only high‐risk NB were mixed in the comparison against MYCN, and also 11q‐deletion was not considered. MNA is exclusively high‐risk and different‐risk subgroups have completely different prognosis as well as biology and genetic alterations. To define the individual influence of 11q‐deletion and MNA in the tumor‐infiltrating immune cell populations exclusively in the high‐risk subgroup, NB patients were divided into 11q‐deleted, MNA, and patients without both alterations. Differential immune proportions between subgroups were also validated in an independent cohort. Validation analysis demonstrated that 11q‐deleted NB displays a higher proportion of CD8+ T cells, M0, M1, and M2 macrophages compared to MYCN‐amplified NB. Additionally, a higher proportion of resting CD4+ memory T cells were also identified in 11q‐deleted NB compared to any of the other two groups.

Total macrophage levels, including resting (M0) and polarized (M1 and M2) states, are increased in 11q‐deleted NB compared to MYCN‐amplified tumors. M1 tumor‐associated macrophages (TAMs) exert an antitumor function through the secretion of pro‐inflammatory cytokines such as IL‐12, tumor necrosis factor (TNF)‐α, CXCL‐10, and interferon (IFN)‐γ and by increasing the levels of nitric oxide synthase (NOS) [33]. In contrast, M2 TAMs suppress the immune response via the secretion of TGF‐β, IL‐10, and arginase 1 and stimulate tumor growth through the secretion of IL‐17, IL‐23, and pro‐angiogenic factors [33]. Hence, the increased proportion of M2 TAMs in the 11q‐deleted subgroup is paving the way to tumor invasion and treatment resistance. We hypothesize that 11q‐deleted NB prime the niche that favors the increased polarization toward M2 macrophages in this subgroup.

Crosstalk between M2 macrophages and Tregs, immunosuppressive factors of the TME (TGF‐β, IL‐10, IDO1), and tumor antigen PD‐L1 cause CD8+ T‐cell inactivation and contribute to inefficient CD8+ T‐cell response priming [34]. This evidence suggests that despite the higher presence of CD8+ T cells in the niche of 11q‐deleted tumors, they may not be correctly activated and thus are unable to mount an effective antitumor immune response.

Resting CD4+ memory T cells are also more abundant in 11q‐deleted NB. Interestingly, the conversion of resting CD4+ T cells into Tregs is dependent on increased levels of TGFβ [35]. We found that TGFβ1 was significantly expressed in the 11q‐deleted tumor, thus probably contributing to tumor immune escape in these patients. Tregs are key immunosuppressive cells that exert their functions by suppressing antigen‐presenting cells via cytotoxic T lymphocyte antigen 4 (CTLA‐4), IL‐2 consumption, and production of immune suppressive cytokines and molecules [36]. However, Tregs are only significantly more abundant in 11q‐deleted NB tumors of the Westermann cohort, but not in the TARGET cohort, perhaps reflecting clinical differences between the German and the American cohorts. Age at diagnosis varies between these two cohorts with high‐risk patients younger than 1.5 years being 28% and 1.9%, respectively. It is also possible that Treg conversion from CD4+ resting T cells may be affected during the course of tumor evolution. These clinical variations between both cohorts may also be affecting activated NK cell levels that appear to be higher in 11q‐deleted subgroup compared to the rest of high‐risk NB tumors in the Westermann cohort, but in the validation cohort they are diminished. This discrepancy needs to be further analyzed in future studies.

Tumor eradication by anti‐GD2 mAbs is mainly based on NK cell‐mediated ADCC [13, 14]. However, an immunosuppressive TME has shown to impair NK cell antitumor activity [37]. Macrophages can also respond to immunotherapy by inhibiting NK cell‐mediated ADCC and T cell‐mediated cytotoxicity in breast cancers and lymphomas through the upregulation of PD‐L1 and IDO1 [38]. Since M2 TAM levels are higher in 11q‐deleted NB, anti‐GD2 immunotherapy may result in enhanced inhibition of NK and cytotoxic T cells in this subgroup, and therefore, they may better benefit from therapeutic antibody plus immune checkpoint blockade by the synergistic effects reported in breast cancers and lymphomas.

Finally, we also observed that there are almost no γδ T cells infiltrating high‐risk NB. γδ T cells are receiving increasing attention due to their function in cancer immunosurveillance and potential for cancer immunotherapy. However, in recent years protumor activities have been linked to γδ T‐cell cells [39]. Dissecting the exact role of γδ T cells in high‐risk neuroblastoma would be interesting to harness its plausible application in this type of tumor.

Our genomic profiling analysis results show that within the high‐risk group, 11q deletion associates with higher SCA, which has been correlated with immune evasion and reduced response to single immunotherapy in other cancer types [40]. On the other hand, the enriched gene ontology analysis revealed that loss of 11q‐located miRNAs has a direct effect on the immune response. Specifically, *miR‐34* is responsible for most of the crosstalk, reflecting its important role in NB pathogenesis. The *mir‐34* family is comprised of three transcripts encoded by two different *loci*. Whereas *miR‐34b* and *miR‐34c* are encoded by the same primary transcript (*miR‐34b/c*), *miR‐34a* is encoded by its own transcript and its location in 1p36 is also frequently deleted in NB [41]. The three miRNAs are transcriptionally regulated by p53 upon DNA damage and their loss has been widely associated with cancer, including NB [31, 42]. As a result, miR‐34‐based therapies are being evaluated in the clinic [43]. Our study also reveals *IL7R* (interleukin 7 receptor) as a miR‐34b/c target gene. Interestingly, McArdle *et al*. [44] detected that interleukin 7 (IL7) was upregulated in 11q‐deleted NB. IL7/IL7R axis regulates the survival and development of memory CD4 cells [45]. Thus, dysregulation of IL7/IL7R axis may be responsible for the immune failure in these tumors and represents a potential therapeutic pathway. Besides, *miR‐4492* is *s*ignificantly downregulated in meningioma cells [46] and its silencing has shown to inhibit FOXK1 expression and promote colorectal cancer proliferation [46]. In contrast, it is overexpressed in breast cancer cells [47], suggesting that its unbalanced expression may regulate the pathophysiology of different tumors. These data, together with the higher expression of immune inhibitory molecules, show that NB patients with 11q deletion create an immunosuppressive microenvironment that could explain why these tumors do not fully benefit from standard anti‐GD2 immunotherapy.

High‐risk NB with 11q deletion represents itself a challenge for immunotherapy as it shows an overall low TMB and tumor‐infiltrating T cells, making it both a poorly immunogenic group and a ‘cold tumor,’ respectively [48]. Hence, a combination of anti‐GD2 with other immunotherapy strategies to circumvent the immunosuppressive phenotype is needed to efficiently promote the immune response in high‐risk 11q‐deleted NB. One approach to overcome tumor escape is the use of immune checkpoint inhibitors (ICIs) to block the inhibitory factors that hamper the host's immune response, such as CTLA‐4, programmed cell death protein 1 (PD‐1), and its ligand, PD‐L1. Treatment with ICIs have shown remarkable clinical benefits in some cancers, but only a fraction of patients respond to treatment [49]. Under physiological conditions, PD‐1/PD‐L1 interaction induces immune tolerance but PD‐L1 overexpression allows tumor cells to evade the host immune system. At the same time, higher PD‐L1 expression has shown to predict increased response to PD‐L1/PD‐1 pathway blockade [50]. Notably, our transcriptomic analysis shows that 11q‐deleted NB display increased levels of PD‐L1 expression compared to patients without it. Therefore, concomitant immunotherapy to release PD‐1/PD‐L1 axis blockade could result in an increased anti‐GD2 response in these patients. Noteworthy, murine models treated with anti‐GD2 in combination with PD‐1 blockade showed a strong reduction of tumor growth, prolonged survival, and the highest cytotoxicity against NB cells [51]. Currently, a phase I study is investigating the combination of ch14.18/CHO with nivolumab (anti‐PD‐1) in children with relapsed NB (NCT02914405). Our results suggest that 11q‐deleted NB will preferentially benefit from this strategy.

The intrinsic genomic instability observed in the 11q‐deleted tumor subset is a weakness that can be therapeutically exploited. Novel evidence links DNA damage defects in tumors with higher immunotherapy response, presumably due to higher neoantigen load eliciting increased T‐cell‐mediated antitumor immune responses [52]. Durable response to ICI therapy has been seen in patients with microsatellite instability (MSI) and higher mutation frequencies in DDR‐related genes [53, 54]. In fact, MSI arising from mismatch repair (MMR) defects is a current criterion for the treatment of solid tumors with anti‐PD‐1 pembrolizumab [55]. However, high TMB and MSI do not always predict favorable responses to ICI, highlighting the dependence of immunotherapy response on other determinants [56].

The effectiveness of several targeted therapies shows that we are entering an era in which treatment decisions will be based on the tumor profile of each patient so that an individualized and molecularly targeted therapy can be applied to each tumor subtype. In order to advance in NB treatment, tumor heterogeneity needs to be considered in treatment decisions and in the rational design of future clinical trials. Several approaches are being tested clinically to revert the inhibitory effect of immunosuppressive mechanisms. Some of these strategies, including antibodies against PD‐L1 [57], IDO inhibitors [58], and Tregs depletion with reagents targeting CD25 [59] have proved clinical activity as cancer immunotherapy. Whether these strategies have a therapeutic impact on 11q‐deleted tumors remains to be determined.

## 5 Conclusions

Our data reveal that high‐risk M stage 11q‐deleted NB displays an intratumoral immunosuppressive signature distinct from other high‐risk subtypes, implicating a mechanism which promotes their escape from immune response. Therefore, high‐risk 11q‐deleted NB patients may preferentially benefit from the combination of anti‐GD2 immunotherapy with immune checkpoint inhibitors (i.e., anti‐PD‐L1, anti‐CTLA‐4, IDO1 inhibitors, etc.) to disable immune escape. Evaluation of this therapeutic strategy is ongoing in open clinical trials and careful evaluation of results for this specific NB subtype will provide valuable insights for future interventions related with precision treatment of 11q NB.

## Conflict of interest

The authors declare no conflict of interest.

## Author contributions

EC, JFdM, AC, VC, and RL conceptualized the study; EC and JFdM involved in methodology; EC, YY, JFdM, AC, and VC made formal analysis; EC, LR, FVS, YY, VC, and JFdM investigated the study; FVS, AC, VC, JFdM, and RL provided resources; EC, JP, and JFdM involved in data curation; EC involved in writing—original draft preparation; JFdM, VC, YY, and EC involved in writing—review and editing; RL and FVS supervised the study; and JFdM acquired the funding.

## Supporting information


**Table S1.** Absolute immune fraction scores in 11‐deleted NB *versus* 11q‐normal NB in the Westermann cohort.

**Table S2.** Absolute immune fraction scores in 11‐deleted NB *versus* 11q‐normal NB in the TARGET cohort.

**Table S3.** Genetic characteristics of the miRNAs located in 11q SRO.

**Table S4.** Gene Set Enrichment Analysis (GSEA) of targeted genes by 11q miRNAs were analyzed with the R package (PGSEA) from BioConductor (http://www.bioconductor.org/biocLite.R). Tab 1 contains the results of the functional profiling using the GO database. Tab 2 contains the enriched biological processes of the 11q miRNAs set targeted genes. Functional blocks from the GO Biological Process (http://www.geneontology.org/) are used in this analysis. A significance level of 0.05 was used. Tab 2 displays the significant and nonredundant terms (N regulated = 11). Response to cytokine (GO:0034097) contains genes that significantly predict cytokine‐mediated signaling pathway (GO:0019221) as well as cellular response to cytokine stimulus (GO:0071345) and thus, the last two pathways are combined in the first one. In the same manner, negative regulation of immune system process (GO:0002683) contains genes shared with the regulation of immune system process (GO:0002682) and is also contained in the first one. Complete targeted genes list and KEGG pathways for each Biological Process is indicated in the subsequent tabs (GO_0006470, GO_0030031, GO_0043043, GO_0002682, GO_0006518, GO_0009617, GO_0045732, GO_0019221, GO_0002683, GO_0071345, GO_0034097).

**Table S5.** Differential expression of genes targeted by the 11q allocated miRNAS based on the Westermann cohort data, exclusively selecting high‐risk NB with 11q deletion *vs*. normal 11q, using R2 genomics analysis and visualization platform (https://r2.amc.nl).

## Data Availability

Westermann and TARGET NB cohorts are accessible at https://r2.amc.nl and https://ocg.cancer.gov/programs/target/projects/neuroblastoma, respectively.
