# Supplementary material for: Intratumoral immunosuppression profiles in 11q‐deleted neuroblastomas provide new potential therapeutic targets
Source: Mol Oncol. 2021 Jan 19;15(2):364–80. doi: 10.1002/1878-0261.12868 (PMC7858123; doi:10.1002/1878-0261.12868)
Supplement: Supplementary file 3 — Table S3. Genetic characteristics of the miRNAs located in 11q SRO. [file MOL2-15-364-s003.docx]

**Supplementary Table 3. Genetic characteristics of the miRNAs located in 11q SRO.**

| **ID** | **Accession** | **Chr** | **Start** | **End** | **Strand** |
| --- | --- | --- | --- | --- | --- |
| [hsa-mir-4491](http://mirbase.org/cgi-bin/mirna_entry.pl?acc=hsa-mir-4491) | MI0016853 | 11 | 111347757 | 111347824 | + |
| [hsa-mir-34b](http://mirbase.org/cgi-bin/mirna_entry.pl?acc=hsa-mir-34b) | MI0000742 | 11 | 111512938 | 111513021 | + |
| [hsa-mir-34c](http://mirbase.org/cgi-bin/mirna_entry.pl?acc=hsa-mir-34c) | MI0000743 | 11 | 111513439 | 111513515 | + |
| [hsa-mir-4301](http://mirbase.org/cgi-bin/mirna_entry.pl?acc=hsa-mir-4301) | MI0015828 | 11 | 113450023 | 113450088 | - |
| [hsa-mir-6716](http://mirbase.org/cgi-bin/mirna_entry.pl?acc=hsa-mir-6716) | MI0022550 | 11 | 118644000 | 118644079 | + |
| [hsa-mir-4492](http://mirbase.org/cgi-bin/mirna_entry.pl?acc=hsa-mir-4492) | MI0016854 | 11 | 118910708 | 118910787 | + |

ID, identifier; hsa, Homo sapiens; mir, microRNA; Chr, Chromosome, coordinates GRCh38.
